# Integrated miRNA Signatures: Advancing Breast Cancer Diagnosis and Prognosis

**DOI:** 10.3390/biom14111352

**Published:** 2024-10-24

**Authors:** Maria Samara, Eleni Thodou, Marina Patoulioti, Antigoni Poultsidi, Georgia Eleni Thomopoulou, Antonis Giakountis

**Affiliations:** 1 Department of Pathology, Faculty of Medicine, School of Health Sciences, University of Thessaly, Biopolis, Mezourlo, 41500 Larissa, Greece; 2 Department of Biochemistry and Biotechnology, University of Thessaly, Biopolis, Mezourlo, 41500 Larissa, Greece; 3 Surgical Department, Faculty of Medicine, University Hospital of Larissa, University of Thessaly, Biopolis, Mezourlo, 41500 Larissa, Greece; 4 Diagnostic Cytopathology Department, Attikon General Hospital, School of Medicine, The National and Kapodistrian University of Athens, 11527 Athens, Greece

**Keywords:** miRNA, breast cancer, biomarker, diagnosis, prognosis, multi-cancer transcriptomics, ROC

## Abstract

Breast cancer ranks first in incidence and second in deaths worldwide, presenting alarmingly rising mortality rates. Imaging methodologies and/or invasive biopsies are routinely used for screening and detection, although not always with high sensitivity/specificity. MicroRNAs (miRNAs) could serve as diagnostic and prognostic biomarkers for breast cancer. We have designed a computational approach combining transcriptome profiling, survival analyses, and diagnostic power calculations from 1165 patients with breast invasive carcinoma from The Cancer Genome Atlas (TCGA-BRCA). Our strategy yielded two separate miRNA signatures consisting of four up-regulated and five down-regulated miRNAs in breast tumors, with cumulative diagnostic strength of AUC 0.93 and 0.92, respectively. We provide direct evidence regarding the breast cancer-specific expression of both signatures through a multicancer comparison of >7000 biopsies representing 19 solid cancer types, challenging their diagnostic potency beyond any of the current diagnostic methods. Our signatures are functionally implicated in cancer-related processes with statistically significant effects on overall survival and lymph-node invasion in breast cancer patients, which underlie their strong prognostic implication. Collectively, we propose two novel miRNA signatures with significantly elevated diagnostic and prognostic power as a functionally resolved tool for binary and accurate detection of breast cancer and other tumors of the female reproductive system.

## 1. Introduction

Breast invasive carcinoma (BRCA) is the most frequent type of cancer in females and the fourth leading cause of cancer-related deaths for both sexes worldwide. In 2022, there were 2.3 million new cases and more than 650,000 deaths. France, Australia, New Zealand, Northern Europe, and the Northern USA exhibit the highest incidence rates, while Fiji Island, Western Africa, and Polynesia display the highest mortality rates, underscoring the geographical heterogeneity of the disease [1]. Female breast cancer is heterogeneous, with distinct molecular subtypes and clinical outcomes [2]. The majority of BRCA cases (85–90%) are sporadic and mainly correlate to environmental risk factors such as lifestyle, obesity, alcohol consumption, and late menopause. However, a percentage of 5–10% of BRCA cases are hereditary and arise from mutations inherited from parents to offspring. *BRCA1/2*, *PTEN*, *ATM*, *CHECK2*, and *PALB2* are the most frequently mutated genes [3].

Adenocarcinomas account for almost 95% of BRCAs, with ductal and lobular adenocarcinomas representing the most frequent subtypes [4]. Comprehensive gene expression profile studies, mainly based on the PAM50 gene signature, have led to the molecular classification of BRCAs. In 2021, the TCGA consortium suggested five groups, namely Luminal A, Luminal B, HER2-enriched, Basal-like, and Claudin-low, based on genomic analysis of 1095 primary BRCA patient samples. However, in their study, they included not only the most frequent histological subtypes but also six rare special histological subtypes. This approach enabled a consensus classification of 12 subgroups, organized into four broader groups of low-, high-differentiation, luminal, and basal [5].

Molecular subtyping is a valuable tool for oncologists as it facilitates patient management, providing them with the most appropriate treatment. Currently, treatment of Luminal A/B tumors expressing hormonal receptors (estrogen, progesterone) relies on endocrine therapy with aromatase or estrogen receptor inhibitors (tamoxifen). HER2-enriched BRCAs, which represent a 15% to 20% percentage of newly diagnosed cases, undergo treatment with chemotherapy or targeted therapy against HER2 activity, such as TKI inhibitors (e.g., Lapatinib) or humanized monoclonal antibodies (e.g., Trastuzumab). Triple-negative BRCA cases are usually treated with chemotherapeutic agents (e.g., paclitaxel, docetaxel), while for BRCA1/2 mutated tumors, PARP inhibitors are recommended (e.g., Olaparib, Talazoparib) [6,7,8].

Notwithstanding significant advances in breast cancer treatment, patients develop resistance to treatment, but the associated mechanisms are still unclear. Within the first three years, 10–15% of BRCAs present metastases. Cellular plasticity and the tumor microenvironment can influence diagnosis and prognosis. Beyond that, the existence of inter- and intra-tumoral heterogeneity weakens current treatment options. The 12 consensus groups proposed by the TCGA group verify the complicated nature of breast cancer, while histological and molecular profiles, genomic and epigenetic alterations, and metabolic changes can explain the currently observed high mortality rates [9].

Early detection is essential for preventing the development of distal metastases and for minimizing mortality rates in all cancer types, including female breast cancer. Mammography is an established technique for early BRCA diagnosis; however, false negative and mainly false positive results can lead to unnecessary biopsies and radiation exposure [10]. The sensitivity of mammography ranges from 62.2% to 89.5%, while specificity approaches 62.7%. Dense breasts and calcified lesions reduce sensitivity and hinder early breast cancer diagnosis. Ultrasound (US) may serve as a supplementary test, but its assessment is influenced both by the equipment and the operator [11]. A recent study comparing the imaging findings from different methods with the subsequent pathological diagnoses concluded that the diagnostic accuracy of mammography, US, and Magnetic resonance imaging (MRI) were 77.9%, 85%, and 86.9%, respectively. MRI showed a sensitivity of 72.2% and US of 61% [12]. Furthermore, invasive biopsy either through a core needle or surgically is painful with possible side effects and unpleasant to patients. Hence, current research focuses on exploring non-invasive biomarkers that can be accurately detected in both tissues and bodily fluids, with prognostic and predictive potential.

Recently, high-throughput techniques have facilitated a comprehensive analysis of genetic and epigenetic profiles in many cancers, thereby expanding our knowledge. miRNAs are small, non-coding molecules with a length of 21 to 24 nucleotides that regulate the expression of genes implicated in cellular processes such as differentiation, proliferation, and carcinogenesis [13]. miRNAs exert “oncogenic” or “tumor suppressor” activities. Up-regulation of oncogenic miRNAs (OncomiRs) or down-regulation of tumor-suppressor miRNAs can lead to breast cancer development. As previously reported, certain miRNAs, such as miR-122, miR-22, and miR-93, exhibit both functions in BRCAs [14,15,16].

Several studies have been focusing on miRNA investigation, assessing their diagnostic, prognostic, and therapeutic potential. According to their results, breast cancer subtypes exhibit specific patterns of miRNA expression [17]. miRNA molecules interplay in various processes such as apoptosis, autophagy, and epithelial-to-mesenchymal transition (EMT) [18]. Previous studies have proposed that up-regulation of hsa-mir-7-5p, hsa-mir-15a, hsa-mir-16, and hsa-mir-17-5p levels can induce apoptosis in breast cancer cells [19,20,21]. Autophagy is implicated in the regulation of tumor recurrence and resistance to cancer treatment. Elevated levels of hsa-mir-20a are negatively correlated with autophagy pathway function and are also linked to a higher incidence of copy number variants and mutations in BRCAs [22]. hsa-mir-26b and hsa-mir-129-5p also inhibit autophagy. Additionally, hsa-mir-129-5p and hsa-mir-200c can reduce the radio-resistance of breast cancer cells. The EMT process is linked to cell mobility and migration and is affected by several up-regulated and down-regulated miRNAs. In breast cancer cells, elevated levels of hsa-mir-23a and hsa-mir-27a are linked to migration and metastatic potential [23,24]. To date, despite the existence of 55 studies registered on the Clinical Trials platform for using miRNAs as diagnostic or prognostic biomarkers in breast cancer, there is no commercially available panel based on miRNAs.

In this study, we present the results of a computational pipeline that relies on a multifaceted analysis of publicly available TCGA data to evaluate miRNA expression levels in terms of diagnostic and prognostic performance for female breast cancer diagnosis. Our analysis provided two molecular signatures consisting of four up-regulated and five down-regulated miRNAs that collectively outperform existing diagnostic methods based on ROC AUC calculations. Both signatures exhibit strong diagnostic and prognostic power and upon clinical validation, could serve as an auxiliary to the existing diagnostic approaches test for breast cancer detection.

## 2. Materials and Methods

### 2.1. Transcriptome Data Acquisition and Preprocessing

miRNA transcriptome data were acquired as normalized miRNA expression values from the GDC Data portal (https://gdc.cancer.gov/, accessed on 8 January 2020). The final complete dataset consisted of 1.165 samples, further subdivided into 90 paired paracancerous biopsies and 181 Stage_I, 614 Stage_II, 245 Stage_III, 22 Stage_IV, and 13 Stage_X tumor samples from male and female patients. miRNA expression data filtering was performed as previously described [25], resulting in the removal of 471 low or non-expressed miRNAs in the BLCA dataset (25% of the total). The remaining 1399 miRNAs were subsequently subjected to downstream analysis as indicated below.

### 2.2. miRNA Transcriptome Analysis and Venn Diagram Comparisons

The filtered raw expression data were cross-normalized and subjected to differential expression analysis with the Bioconductor package edgeR (https://bioconductor.org/packages/release/bioc/html/edgeR.html, v4.2.2, accessed on 21 February 2024, [26]) in R. The analysis was focused on the following comparisons: (i) Paracancerous vs. all tumors, (ii) Paracancerous vs. Stage_I tumors, (iii) Paracancerous vs. Stage_II tumors, (iv) Paracancerous vs. Stage_III tumors, and (v) Paracancerous vs. Stage_V tumors. The applied thresholds selecting the differentially expressed miRNAs were FDR < 0.05 and log2FC > 1 or <−1 for tumor up-regulated or tumor down-regulated miRNAs, respectively. Volcano plots were prepared with the EnhancedVolcano Bioconductor package in R (https://bioconductor.org/packages/release/bioc/html/EnhancedVolcano.html, v1.22.0, accessed on 21 February 2024). Venn diagrams were also performed in R.

### 2.3. Heatmap Construction

The Bioconductor ComplexHeatmap package [27] was used to generate heatmap illustrations of normalized (log2 and z-score transformed) miRNA signature expression in BRCA samples or across all cancer types from the multi-cancer panel according to previously described arguments [25].

### 2.4. ROC Analysis of miRNA Expression in TCGA BRCA

All remaining miRNAs from the TCGA BRCA dataset after filtering were subjected to ROC analysis using the R package EasyROC [28]). The Optimal Cutpoint package [29] was used for the cut-off analysis with the application of previously reported selection criteria [25]. All ROC plots were generated in R.

### 2.5. Box Plot Analysis of miRNA Expression Across Clinical Manifestations

Patients were stratified according to three clinical manifestations of BRCA (pathological t stages T1–T4 and TX, AJCC regional lymph node status N0–Nx, AJCC distant organ metastasis M0-Mx) according to the associated clinical information. ANOVA Holm–Sidak test followed by multiple comparisons analysis was performed with SigmaPlot v11 https://alfasoft.com/software/statistics-and-data-analysis/data-visulization/sigmaplot/, accessed on 23 February 2024). Box/dot plots were generated with ggplot2 (https://ggplot2.tidyverse.org/, accessed on 23 February 2024) in R.

### 2.6. miRNA Target Network and Functional Enrichment Analysis

miRNET [30] was used to generate the miRNA target network, using the miRbase IDs of both miRNA panels as input for network construction and considering the miRTarBase v8 genes for target prediction. Functional analysis was performed with KEGG and DisGeNET through hypergeometric enrichment.

### 2.7. Kaplan Meier Analysis of miRNA Signatures

The R RTCGA package [31] was used for prognostic evaluation of both miRNA signatures in the form of Kaplan–Meier analysis in R. Patient stratification was performed through the calculation of the optimal cutpoint for each miRNA signature through the survival cutpoint function, while statistical analysis was based on logrank *p*-value calculation for overall survival and lymph node invasion as previously described [25]. Prognostic analysis results were visualized with ggplot in R.

## 3. Results

### 3.1. miRNA Transcriptome Analysis in the TCGA Breast Cancer Patient Cohort

**Figure 1:**
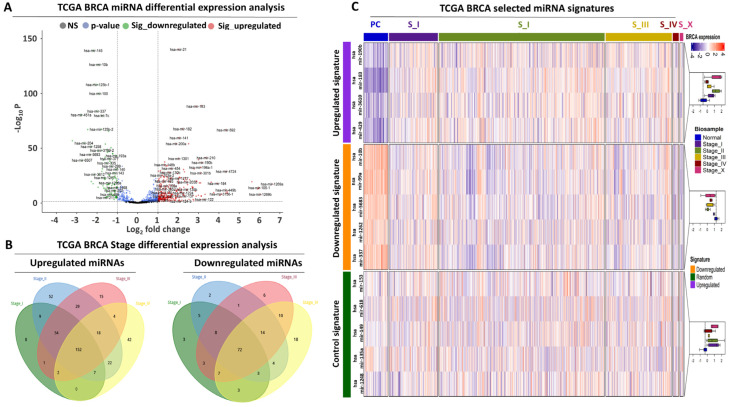
**miRNA transcriptome analysis in breast cancer patients (TCGA-BRCA cohort)** (**A**) Volcano plot summarising the up-regulated (red) and down-regulated (green) miRNAs between cancerous (*N* = 1075) and paracancerous (*N* = 90) breast biopsies. Dashed lines indicate statistical significance cutoffs (horizontal) or >1/<−1 log2 fold change cutoffs (vertical) respectively. (**B**) Venn diagrams highlighting the commonly up- (left) or down-regulated (right) miRNAs across all breast tumor stages. (**C**) Heatmap analysis illustrating the selected up- or down-regulated miRNA signatures across all BRCA patient biopsies. The control miRNA panel, consisting of the hsa-mir-153, hsa-mir-618, hsa-mir-149, hsa-mir-135a, and hsa-mir-1248, is also shown for comparison. Boxplots on the right summarize the expression of each miRNA signature across all BRCA biosamples.

Differential expression analysis was performed with the aim of isolating miRNAs that significantly alter their expression in breast tumors compared to paracancerous mammary tissue. The analysis utilized 1165 biopsies from the breast carcinoma (BRCA) patient cohort of The Cancer Genome Atlas (TCGA) (Supplementary Table S1). Initially, all-staged tumors were compared against the paracancerous biopsies, revealing 413 differentially expressed miRNAs. Interestingly, the majority (73.8%) of these differentially expressed miRNAs increased their expression in cancerous compared to paracancerous tissues (Figure 1A, Supplementary Table S2).

We independently performed differential expression analysis for each tumor stage against the paracancerous samples to investigate miRNA expression changes across disease progression. Venn analysis revealed that the majority of the significantly up- or down-regulated miRNAs were commonly and unidirectionally affected across all tumor stages (Figure 1B). This observation suggests that the regulatory events that drive miRNA expression are not only established early upon malignant transformation but are stably maintained during neoplastic progression. Based on the results of the miRNA transcriptome analysis, we selected two miRNA panels consisting of four commonly up-regulated (hsa-mir-190b, hsa-mir-183, hsa-mir-3610, and hsa-mir-429) and five commonly down-regulated (hsa-mir-10b, hsa-mir-99a, hsa-mir-5683, has-mir-1262, and hsa-mir-337) miRNAs across all tumor stages (Figure 1C, Supplementary Table S3), which we evaluated further in terms of diagnostic power in BRCA patients.

### 3.2. Evaluation of the Diagnostic and Prognostic Power of the Up-Regulated miRNA Signature

**Figure 2:**
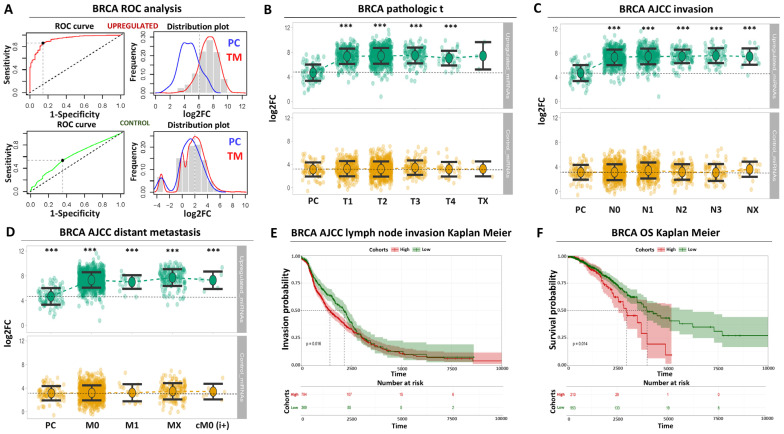
**Diagnostic and prognostic properties of the up-regulated miRNA signature in breast cancer patients** (**A**) ROC analysis for the up-regulated (red curve) and the control miRNAs (green curve). Upper left plot indicates ROC AUC performance for separating tumor and paracancerous biopsies. The upper right plot demonstrates the distribution of miRNA expression in tumor (TM, shown in red) or paracancerous (PC, shown in blue) biopsies. The plots at the bottom correspond to the AUC or distribution of miRNA expression for the control miRNAs. (**B**) Beeswarm plot illustrating the expression of the up-regulated miRNAs (upper plot) across all tumor stages compared to paracancerous levels. Control miRNAs (bottom plot) are also shown for comparison. Horizontal dashed line indicates the average of paracancerous expression (**C**) Same as (**B**) for AJCC lymph node invasion (**D**) Same as (**C**) for AJCC distant organ metastasis (**E**) Kaplan–Meier analysis comparing lymph node invasion between patients with high (red curve) and low levels (green curve) of miRNA signature (log2rank = 0.016). (**F**) Same as (**E**) for overall survival (OS, log2rank = 0.014). Asterisks in all graphs indicate the level of statistical significance based on ANOVA (***: *p*-value < 0.001).

We subjected the up-regulated miRNA signature to ROC analysis designed to evaluate its collective diagnostic potential for tumor biopsies compared to paracancerous breast tissues. The AUC performance of the up-regulated panel was 0.92 (CI: 0.89–0.95), followed by 0.89 sensitivity and 0.85 specificity (Figure 2A, Supplementary Table S4). In terms of individual miRNA performance, hsa-mir-3610 had the lowest AUC (0.79) in contrast to hsa-mir-183, which was associated with the highest AUC score (0.95) among the four up-regulated miRNAs (Supplementary Table S4). In sharp contrast, the collective ROC performance of the control miRNA signature was lower (AUC = 0.52, sensitivity = 0.53, specificity = 0.64) and not statistically significant (Figure 2A, Supplementary Table S5). As expected, the elevated discriminatory power of the up-regulated miRNA panel compared to the control miRNAs was strongly reflected in the distribution of miRNA expression among paracancerous and tumor BRCA samples, effectively combining the differential expression properties with ROC performance for these miRNAs (Figure 2A). In conclusion, ROC analysis revealed that the up-regulated miRNA panel is associated with high and statistically significant diagnostic power for breast tumors compared to paracancerous tissues.

Beyond diagnostic performance, we profiled the expression of the up-regulated miRNA signature across multiple clinical manifestations of the disease, challenging its prognostic potential. Initially, we confirmed that our selected miRNAs are significantly up-regulated in all tumor stages compared to paracancerous levels even when a different tumor staging system is considered (Figure 2B, Supplementary Table S6). We dissected further the apparent connection between miRNA expression and tumor status to observe that these miRNAs are significantly expressed in all stages of lymph node invasion (Figure 2C, Supplementary Table S6) or distant organ metastasis (Figure 2D, Supplemantary Table S6) compared to paracancerous biopsies. Of note, the expression of the control miRNAs remained unchanged between tumor and paracancerous tissues, regardless of tumor stage, invasion status, or distant organ metastasis (Figure 2B–D). Importantly, high miRNA levels in breast tumors were significantly associated with the shortening of disease progression time, as manifested through Kaplan–Meier analysis for lymph node invasion (Figure 2E). As a result, elevated levels of the up-regulated miRNA panels were associated with a significant shortening of overall survival time for breast cancer patients (Figure 2F). Taken together, these data indicate that our selected up-regulated miRNA signature, apart from an enhanced diagnostic potential, also exhibits significant prognostic power for breast cancer patients. In addition, these observations suggest that up-regulation of these miRNAs occurs during the early stages of the disease and persists during all subsequent stages of breast tumor progression.

### 3.3. Evaluation of the Diagnostic and Prognostic Power of the Down-Regulated miRNA Signature

**Figure 3:**
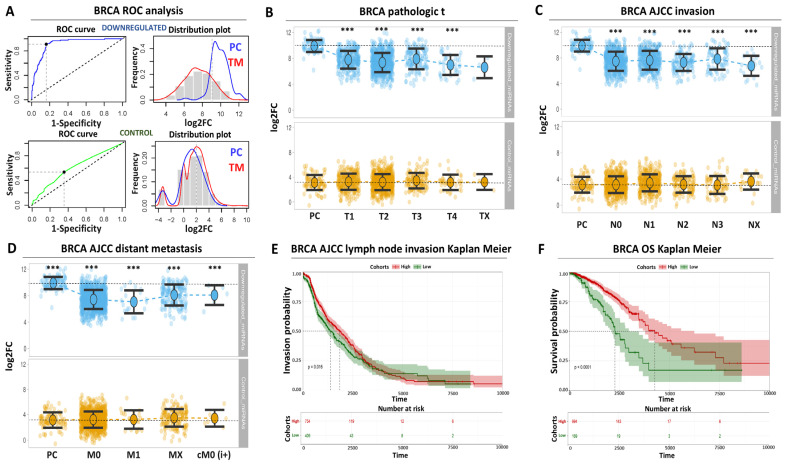
**Diagnostic and prognostic properties of the down-regulated miRNA signature in breast cancer patients** (**A**) ROC analysis for the down-regulated (blue curve) or control (green curve) miRNAs. Upper left plot indicates ROC AUC performance for separating between paracancerous and tumor breast tissues. The upper right plot demonstrates the distribution of miRNA expression in tumor (TM, shown in red) or paracancerous (PC, shown in blue) biopsies. The plots at the bottom correspond to the AUC or distribution of miRNA expression for the control miRNAs. (**B**) Beeswarm plot illustrating the expression of the down-regulated miRNAs (upper plot,) across all tumor stages compared to paracancerous levels. Control miRNAs (bottom plot) are also shown for comparison. Horizontal dashed line indicates the average of paracancerous expression (**C**) Same as (**B**) for AJCC lymph node invasion (**D**) Same as (**B**) for AJCC distant organ metastasis (**E**) Kaplan–Meier analysis comparing lymph node invasion between patients with high (red curve) and low levels (green curve) of miRNA expression (log2rank = 0.016) (**F**) Similar to (**E**) for overall survival (OS, log2rank = 0.014). Asterisks indicate the level of statistical significance based on ANOVA (***: *p*-value < 0.001).

Having demonstrated the significant diagnostic and prognostic association of our up-regulated miRNA panel for breast cancer, we subsequently focused on the down-regulated miRNA signature. This time we challenged through ROC analysis the down-regulated miRNAs for discrimination of the paracancerous tissues against all tumor stages. The down-regulated signature was associated with a ROC AUC of 0.91 (CI: 0.89–0.93) along with 0.91 sensitivity and 0.83 specificity for breast paracancerous tissues (Figure 3A, Supplementary Table S7).

With regards to individual miRNA performance, hsa-mir-99a was associated with the highest AUC score (0.90), while the lowest ROC AUC performance was observed for hsa-mir-1262 (0.77). Again, we observed a good correlation between an enhanced ROC performance and a higher distribution of miRNA expression in paracancerous tissues compared to breast cancer tumors or the random miRNAs (Figure 3A). To summarize, these results confirm that the selected down-regulated miRNA panel is associated with an elevated diagnostic power against paracancerous breast tissues.

In agreement with the up-regulated miRNA analysis, the observed diagnostic performance of the down-regulated panel was accompanied by significant prognostic power. Expression of this miRNA signature was significantly reduced in all breast tumor stages (Figure 3B, Supplementary Table S6) and was maintained low in breast tumors regardless of lymph node invasion (Figure 3C, Supplementary Table S6) or distant organ metastasis status (Figure 3D, Supplementary Table S6). Furthermore, patients with low levels of these miRNAs are more likely to be associated with an increased likelihood for advanced disease progression manifested in the form of lymph node invasion (Figure 3E) or poor disease outcome in the form of shortening of overall survival time (Figure 3F). Collectively, these observations support the notion that both the up- and down-regulated miRNAs hold great promise as diagnostic and/or prognostic biomarkers for breast cancer.

### 3.4. Multi-Cancer Analysis of Both miRNA Signature Panels

For us, an important prerequisite for creating a successful biomarker signature is the specialization of its presence and/or function against a certain disease, and miRNAs are well known for their tissue- and cancer-specific expression properties. To challenge the performance of our selected signatures beyond breast cancer, we took advantage of our access to a diverse panel of miRNA transcriptomes representing TCGA patients from nineteen different forms of cancer. This analysis allowed us to compare the expression of both selected miRNA panels or the control miRNAs in more than 7000 tumor and paracancerous biopsies organized according to organ primaries into reproductive, uropoetic, thoracic, gastrointestinal, and other.

**Figure 4:**
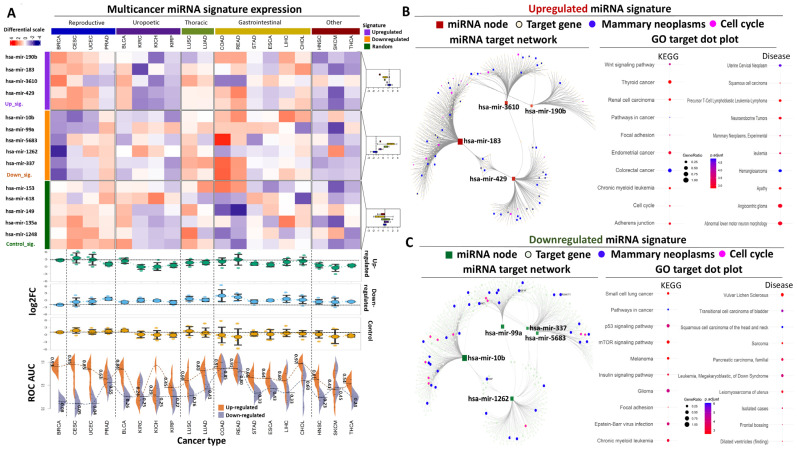
**miRNA multi-cancer expression analysis and target network construction** (**A**) Heatmap comparing normalized fold change for both miRNA signatures or the control miRNAs across nineteen cancer types. Biopsies are shown according to organ primaries. Box plots on the right summarize miRNA signature expression. Dot plots below the heatmap demonstrate average miRNA expression in each cancer (horizontal dashed line indicates average miRNA expression in BRCA). Beanplots at the very bottom, summarize ROC AUC performance of both signatures across each cancer. Numbers indicate average AUC (or 1-AUC) for up- and down-regulated signatures, respectively, while dashes inside each bean highlight AUC of individual miRNAs. (**B**) miRNA target network and GO analysis for the up-regulated signature. Red squares represent individual miRNAs. Blue and magenta nodes highlight targets involved in mammary neoplastic and cell cycle-related processes, respectively. Dot plots highlight the top 10 most significant KEGG pathways or diseases that are enriched for the miRNA targets. (**C**) Same as (**B**) for the down-regulated miRNA signature. Green nodes correspond to the selected down-regulated miRNAs.

Focusing on the up-regulated miRNAs, their average fold change between tumors and paracancerous tissues was higher in BRCA patients (log2FC = 2.89) compared to all other cancer types, except for cervical squamous cell carcinoma and endocervical adenocarcinoma (CESC) in which miRNA fold expression change (log2FC = 3.0) was slightly higher (Figure 4A). This collective pattern of expression was largely reflected also in the individual miRNAs that constitute the up-regulated signature. hsa-mir-3610 was associated with the best individual performance for BRCA across all cancer types, while hsa-mir-429 was associated with reduced specialization since six other types of cancer demonstrated higher tumor/precancerous fold changes for this particular transcript compared to BRCA. Interestingly most miRNAs were upregulated in related cancers of the female reproductive system (CESC, uterine corpus endometrial carcinoma, UCEC) but not in prostate cancer. Beyond the reproductive system, the same miRNAs were associated with elevated tumor/paracancerous fold changes in several gastrointestinal malignancies, while patients with uropoietic forms of the disease consistently demonstrated reduced levels of tumor miRNA expression compared to paracancerous levels. In addition, a comparison of the tumor fold change for the up-regulated panel against the control miRNAs was statistically significant in BRCA or all other tumors of the female reproductive organs but not in the remaining primaries, with the exception of the gastrointestinal tumors, highlighting increased specialization for the female reproductive forms of the disease (Supplementary Table S8).

With regards to the down-regulated miRNA panel, the multi-cancer expression analysis revealed a consistently strong down-regulation in BRCA tumors compared to the remaining forms of the disease. The average fold change (log2FC = −1.96) of this miRNA panel in the BRCA cohort was the lowest compared to all other cancer types, highlighting its specificity for normal mammary epithelium. This collective down-regulation was tightly observed also for the individual miRNAs, with hsa-mir-5683 demonstrating the strongest reduction in tumor expression levels (log2FC = −2.35) and hsa-mir-1262 the weakest (log2FC = −1.38) in BRCA tumors (Figure 4A). In contrast to the up-regulated miRNAs, we did not detect a uniform trend of down-regulation in the remaining cancer types of the female reproductive organs, except for hsa-mir-10b and hsa-mir-99a, the expression of which was equally reduced across BRCA, CESC, and UCEC tumors. Of note, statistical comparison of the fold expression change for this miRNA signature against the control miRNAs did not reveal any significant effects apart from BRCA or the reproductive primaries, indicating a strong discriminating preference for the female reproductive tumors.

Moving beyond differential expression, we also calculated the diagnostic power of both signatures across all tumors (Figure 4A). This analysis revealed exceptionally high AUC (or low 1- AUC) values for the up- or down-regulated miRNAs, respectively, in breast, cervical, and uterus tumors compared to their normal counterparts. Importantly, the spread in average AUC performance between the up- and down-regulated miRNAs in all other tumors was dramatically reduced, suggesting that the combined use of both miRNA panels in female reproductive tumors outperforms their diagnostic power in all other cancer types. For example, our up-regulated miRNAs were associated with high AUC values in other tumor types (e.g., colorectal), but the down-regulated miRNAs failed to accurately detect the corresponding paracancerous biopsies in the same cancers. The exact opposite was observed in kidney tumors (Figure 4A). Taken together, the results from the multi-cancer expression analysis suggest that although both miRNA panels can be individually used for detecting other forms of the disease, their combination dramatically specializes the diagnostic power for breast and/or female reproductive cancers.

### 3.5. Network and Gene GO Analysis of miRNA Targets

Functional insights not only underline the deregulated expression of candidate transcripts but strengthen their role as putative biomarkers for disease onset. Moreover, changes in miRNA expression frequently dictate post-transcriptional degradation of target genes, the function of which governs normal homeostasis or pathological manifestations. To better understand the functional role of our miRNA panels in breast cancer, we subjected both signatures to target mRNA prediction coupled with network and Gene Ontology (GO) analysis.

With regards to the up-regulated miRNAs, hsa-mir-183 was predicted to affect the largest number of target transcripts (351), followed by hsa-mir-429 (151), hsa-mir-3610 (118), and hsa-mir-190b (62) (Figure 4B). Interestingly, hsa-mir-429 shared some of its target genes with the remaining miRNAs that constituted the up-regulated panel, predominantly with hsa-mir-183. Concerning molecular function through mRNA targeting, GO analysis, summarized in the form of KEGG pathways or enriched diseases, revealed extensive enrichment for cancer-associated cellular processes such as cell cycle, apoptosis, p53-mediated regulation, and ErbB signaling (Supplementary Table S9). Importantly, functional enrichment was observed for pathways associated with female reproductive neoplastic functions (e.g., mammary, uterine, cervical, endometrial) and interestingly also for intestinal forms of the disease. This observation is in agreement with the results of the multi-cancer expression analysis according to which several of the selected up-regulated miRNAs were differentially expressed in gastrointestinal-related tumors and not only in BRCA or reproductive neoplasms. It should be noted that several of the commonly targeted mRNAs from multiple miRNAs of the network were also involved in cancer-related processes, indicating that these miRNAs are up-regulated early during disease progression to collectively regulate downstream target genes that in turn control critical processes during malignant transformation.

Focusing on the down-regulated panel, hsa-mir-10b had the largest number of predicted target transcripts (323), followed by hsa-mir-1262 (165), hsa-mir-99a (133), hsa-mir-5683 (100), and finally hsa-mir-337 (81) (Figure 4C). Similar to what was observed for the up-regulated panel, we again observed several common targets among the down-regulated miRNA signature, especially between hsa-mir-10b and hsa-mir-1262 or between hsa-mir-1262 and the remaining four miRNAs. Moreover, these miRNAs were predicted to regulate downstream target transcripts with cancer-related functions, including but not limited to cell cycle arrest, DNA damage, and integrity checkpoints, as well as regulation of cellular migration (Supplementary Table S10). In terms of KEGG or disease pathway, we observed the enrichment of targets involved in various cancers or cancer-related signaling processes such as Wnt, VEGF, ErbB signaling, mammary neoplasm, and endometrial cancer, among others. In conclusion, this analysis revealed that altered expression of the selected miRNA signatures affects cancer-related processes through downstream target regulation, providing a functional basis for their elevated and specialized diagnostic/prognostic properties for breast cancer.

## 4. Discussion

Breast cancer is a major health issue worldwide. Despite recent developments in patient treatment, breast tumors are responsible for approximately 16% of cancer-related deaths globally, mainly due to metastatic onset. The number of new breast cancer cases is rising rapidly, highlighting the urgency of implementing alternative strategies for early diagnosis. So far, X-ray mammography is considered the golden standard for BRCA diagnosis. However, as previously mentioned, its sensitivity and specificity performance are far from perfect [11]. Ultrasonography (US) and magnetic resonance imaging (MRI) can be used as auxiliary methods but are not suitable for small mass or atypical tissue or wide-scale screening and breast cancer staging, respectively [32]. Thus, to improve clinical outcomes and prolong the overall survival of BRCA patients, research should focus on the identification of molecular biomarkers with elevated diagnostic and prognostic potential, which can be easily and accurately detected in tissues or body liquids.

Early diagnosis is crucial for effectively categorizing individuals for treatment and for decreasing mortality rates. Serum biomarkers such as CA 15-3 and CA 27-29 have been approved by the FDA; however, due to their low diagnostic performance (with ROC AUC ranging between 0.60–0.87 for CA27.29 and 0.56–0.85 for CA15.3 [33]), they are mainly used for monitoring breast cancer [34]. Elevated CA-27-29 levels in serum may indicate disease progression or a high tumor load, but specificity is low as well [35,36]. In a more recent clinical study, the authors measured the velocity of CA 15-3 and CEA change over time. They showed that if the velocity of both markers exceeded certain cut-off point values, disease recurrence could be predicted with sensitivity, specificity, negative predictive value, and positive predictive value of 94.0%, 73.1%, 92.5%, and 77.8%, respectively [37]. Contrast-enhanced mammography (CEM) with the aid of computer analysis can classify patients with breast cancer with an ROC curve of about 0.848, while the combination of digital breast tomosynthesis (DBT) with computational analysis is associated with an ROC AUC performance of 0.841 to 0.850 (95% CI, −0.012 to 0.030) [38,39]. Taken together, both of our miRNA signatures outperform these classical diagnostic approaches based on ROC AUC analysis in solid biopsies. In addition, they show better performance as prognostic biomarkers in terms of specificity, compared to the CA 15-3 and CEA velocity change study [37].

In terms of liquid biopsy, a growing body of evidence has demonstrated the diagnostic, prognostic, and predictive significance of miRNAs in breast cancer. However, a commercially accessible miRNA panel is currently unavailable. A recent study extracted data from TCGA and analyzed the miRNA profile of 755 BRCA tissues and 86 paracancerous tissue samples. In this study, 28 differentially expressed (nine up- and nineteen down-regulated) miRNAs were identified in breast cancer tissues versus non-cancerous samples. ROC analysis was performed to evaluate the diagnostic impact of these miRNAs. The AUC values for all 28 miRNAs ranged from 0.83 to 0.99. The combination of the top five miRNAs (hsa-miR-139, hsa-mir-21, hsa-miR-96, hsa-miR-183, and hsa-miR-10b) showed high sensitivity and specificity (96.95% and 100%, respectively) with an AUC value of 0.98, suggesting high diagnostic potential of this miRNA signature [40]. It is worth noting that in our study, we also included hsa-miR-183 and hsa-miR-10b as miRNAs with high diagnostic power, confirming the previously reported results. In both studies, hsa-miR-183 and hsa-miR-10b as miRNAs are up- and down-regulated, respectively, and their expression levels remain unaffected throughout different stages. The differences in AUC values could be due to the different number of samples analyzed or the different algorithms for ROC analysis. It should be noted however that our strategy further expands the previous reports since it is not focused only on breast cancer diagnosis, demonstrating that our proposed up-regulated signature has strong prognostic potential and is associated with an increased specialization for female reproductive cancer types based on a multi-cancer analysis.

A recent study demonstrated that hsa-miR-21 and hsa-miR-10b can serve as early diagnostic biomarkers in the Egyptian female population. In their study, serum miRNA levels were elevated in patients versus controls. ROC analysis revealed an AUC of 0.991 with 97.1% sensitivity and 100% specificity for hsa-miR10b and an AUC of 0.965 with 95.7% sensitivity and 85% specificity for hsa-miR21. However, no other analyses were performed [41]. In a separate study, plasma samples of 226 BRCA patients and 146 healthy individuals were investigated for miRNA diagnostic biomarkers. ROC analysis was performed revealing AUC values ranging between 0.809 and 0.962, which were validated for each one of the nine selected miRNAs. The combination of hsa-miR-1246, hsa-miR-206, hsa-miR-24, and hsa-miR-373 yielded an AUC of 0.992 with an accuracy of 97%, specificity of 96%, and sensitivity of 98% in the validation set, supporting a high diagnostic value for breast cancer detection [42], confirming our strategy for combining miRNAs into molecular signatures.

In another study published in 2021, the diagnostic utility of hsa-miR-25-3p, hsa- miR-29a-5p, hsa-miR-105-3p, hsa-miR-181b1-5p, hsa-miR-335-5p, and hsa-miR-339-5p was examined in 50 paired (cancerous, non-cancerous) BRCA samples. The calculated AUC performance ranged from 0.77 to 0.84 for all miRNAs analyzed (all *p*-values < 0.0001; 95% of CI), suggesting that each of them could serve as a biomarker for breast cancer diagnosis with high sensitivity and specificity [43]. In the same year, analysis of blood samples from 20 females with breast cancer and 20 healthy individuals showed that three miRNA molecules (hsa-miR-21, hsa-miR-155, and hsa-miR-R125) with AUC values of 0.699, 0.856, and 0.846, respectively, could serve as diagnostic biomarkers with an average sensitivity and specificity of 79.6% and 70.6%, respectively [44]. The low sensitivity and specificity values in this study could be explained by the small number of examined cases.

Shimomura et al. (2016) analyzed more than 1280 serum samples from breast cancer patients extracted from the National Cancer Center Biobank in Japan, and more than 2800 females with no cancer (controls). They presented a five-miRNA signature (hsa-miR-1246, hsa-miR-1307-3p, hsa-miR-4634, hsa-miR-6861-5p, and hsa-miR-6875-5p) that could detect breast cancer with an AUC value of 0.971 (accuracy 89.7%, sensitivity 97.3%, and specificity 82.9%) [45]. Their reported diagnostic performance is superior to ours; however, our proposed panel consists of two versatile and highly performing miRNA signatures, suitable for independent detection of breast neoplastic and normal epithelium, providing a binary diagnostic platform that we further challenged across multiple cancer types. Several other miRNAs are also suggested in additional studies; however, their diagnostic/prognostic power was not calculated [46].

In a recent review report, several studies describing a variety of miRNAs with diagnostic, prognostic, and therapeutic potential were included [47]. Eight studies focus on the diagnostic and/or prognostic impact of miRNA signatures, although AUC values and sensitivity plus specificity percentages are calculated and presented in only six of them. It is worth noting that all studies except one are performed on blood samples and not tissue samples. A case-control study conducted by Orange and Motovali-Bashi on tissue samples from female breast cancer patients and controls showed that hsa-miR-9 and hsa-miR-34a could serve as diagnostic biomarkers with AUC values of 0.71 and 0.72 (sensitivity 83.33% and 72%, specificity 70.37% and 76%, respectively) [48]. Neither miRNA was selected in our panels since we set a relatively high AUC threshold for both of our miRNA panels. In the remaining studies performed primarily on blood samples, various miRNA signatures were suggested. In these studies, AUC values ranged from 0.71, the lowest, to 0.941, a performance that broadly agrees with our observations. Sensitivity and specificity percentages ranged from 81.1% to 92.65% and from 74% to 92.31%, respectively [49,50,51,52,53].

In an up-to-date case-control study conducted by Miranda et al. (2024), serum expression levels of the miRNAs hsa-miR-210, hsa-miR-195, hsa-miR-34a, hsa-miR-16, hsa-miR-21, hsa-miR-10b, and hsa-miR-1 were evaluated for diagnostic purposes. All miRNAs examined were downregulated in the serum of BRCA patients against controls. The diagnostic value of this miRNA signature was assessed via ROC analysis. Four miRNAs (hsa-miR-195, hsa-miR-210, hsa-miR-21, and hsa-miR-16) had the best diagnostic performance with a combined AUC value of 0.898 (0.765–0.970), sensitivity of 71.4%, and specificity of 100.0%. Combinations of two or three miRNAs were also tested, revealing collectively higher AUC values compared to individual miRNA performance, again in agreement with our observations. However, the prognostic value of this miRNA signature, along with functional analysis or multi-cancer expression performance, was not performed [54].

Our data reveal a strong diagnostic and/or prognostic potential for both of our suggested miRNA signatures. In 2020, Sang et al. identified a three-miRNA panel with prognostic potential. They extracted raw data and clinical information for 1062 breast cancer samples and 104 non-cancerous samples (TCGA website), resulting in 67 up- and 17 down-regulated miRNA molecules. Nevertheless, three of them, miRNAs hsa-miR-105-1, hsa-miR-301b, and hsa-miR-1258 were linked to overall survival (OS). More specifically, all were correlated with ER and PR hormonal receptors, hsa-miR-105-1 was correlated with hormonal receptors ER and PR, while hsa-miR-301b and hsa-miR-1258 were also linked to age, stage, and/or metastasis [55]. Our analysis confirmed the differential expression of these miRNAs, yet we did not prioritize any of them for our selected signature panels due to their ROC AUC performance.

Another computational study performed by Tian et al. (2021) focused on the prognostic and predictive role of a TCGA-validated signature consisting of five miRNAs in 962 breast cancer patients. Based on Cox regression analysis, the optimum miRNA panel for disease-specific survival (DSS) and OS was the combination of hsa-miR-574, hsa-miR-30b, hsa-miR-224, hsa-miR-210, and hsa-miR-130a molecules. ROC analysis was performed to assess the predictive power of this miRNA panel, with an AUC performance not exceeding 0.679 (95% CI 0.574–0.783) in the validation set [56]. Additionally, in the study of Davarinejad et al. (2022), an analysis of almost 7000 breast cancer patients’ data (GEO, TCGA, patients) revealed a six-miRNA signature (hsa-miR-151a-5p, hsa-miR-34a-5p, hsa-miR-1307-3p, hsa-miR-450b-5p, hsa-miR-501-3p, and hsa-miR-532-5p) with a diagnostic and predictive role. AUC values for diagnostic strength ranged from 0.67 for hsa-miR-34a-5p) to 1.00 for hsa-miR-1307-3p (available as a preprint at https://doi.org/10.21203/rs.3.rs-1551331/v1).

Our study lacks clinical confirmation, yet some of our identified miRNAs have been individually detected in tissue and plasma specimens from patients with breast cancer. In a recent study, 196 matched tissue samples were evaluated and hsa-miR190b was also overexpressed in breast tumors and correlated to ER+ status [57]. Previous studies also support the overexpression of hsa-mir-183 in breast cancer patients. hsa-mir-183 is a component of the miR-183/182/96 cluster, which was upregulated in breast cancer tissue clinical specimens against normal tissue specimens. This cluster was also correlated to TNM stage, distal metastasis, and poor clinical outcomes, supporting a potential role in breast tumorigenesis [58]. Up-regulation of the miR-183/182/96 cluster is also supported in another study performed by Li et al. (2014) and seems to be linked with cell proliferation and migration of breast cancer cells [59]. Cava C et al., in their study (2020) using ten samples of HER2+ BC human tissue and corresponding non-cancerous samples, showed that hsa-mir-429 was upregulated in tumor samples, supporting a diagnostic role, especially for HER2-positive BC patients [60]. We did not find any study that performed in clinical breast cancer samples for hsa-mir-3610.

Some of the down-regulated miRNAs proposed in our study have also been solely detected in clinical samples. In a recent study by Turkoglu F. et al. (2024), serum specimens of 35 breast cancer females and 35 healthy individuals were examined for several miRNAs, including miR10b and hsa-miR99a. In this study, hsa-miR-99a levels were decreased in patients’ serum samples compared to healthy individuals, supporting a potential protective role of hsa-miR-99a in breast cancer [61]. However, hsa-miR10b showed similar expression levels between the two groups. Yet, in a different study conducted by Khalighfard et al. (2018), plasma expression levels of hsa-miR10b were decreased after the operation, chemo- and radiotherapy, in agreement with our observations [62]. Another study showed that hsa-miR-99a-5p expression levels were downregulated in breast cancer tissues compared to normal breast samples, again in alignment with our analysis in solid biopsies; however, plasma levels were upregulated in breast cancer patients [63]. Finally, another study by Kong et al. (2021) showed that hsa-miR-337-3p shows lower expression levels in breast cancer tissues and acts as a tumor suppressor in this type of cancer [64]. So far, there are no validation studies on clinical specimens, tissue, or plasma samples, for the remaining miRNAs supported in our signatures. Taken together, these data reinforce the presence of our selected miRNAs in clinical samples (tissues and/or body fluids) of breast cancer patients.

Target prediction coupled with functional analysis revealed that among the up-regulated miRNAs in our study, hsa-mir-183 affects the largest number of target transcripts, followed by hsa-mir-429, hsa-mir-3610, and hsa-mir-190b. In the down-regulated miRNAs, hsa-mir-10b had the largest number of predicted target transcripts, followed by hsa-mir-1262, hsa-mir-99a, hsa-mir-5683, and hsa-mir-337. Common target genes are mainly shared between hsa-mir-10b and hsa-mir-1262. Importantly, the target genes from both signatures are involved in cancer-related processes such as cell cycle, apoptosis, p53 and DNA damage pathways, ErbB signaling, and cellular migration, providing functional insights that support the observed diagnostic and prognostic performance of our miRNA panels and confirming the selection criteria of our analytical strategy.

Previous studies highlight the functional implications of several miRNAs in cancer metastasis and treatment. With regards to the tumor-expressed panel, hsa-mir-183 regulates the PI3K/AKT signaling pathway, exerting its oncogenic activity through PTEN inhibition, interfering with the cell cycle, BRCA cell proliferation, and migration. It also seems to play a role in breast cancer anti-miR-mediated treatment [65,66]. A systematic review and meta-analysis studying 188 miRNAs showed that upregulation of hsa-miR-183-5p and downregulation of hsa-miR-10b-5p could confer chemoresistance against doxorubicin, an anthracycline antibiotic drug that is used for treatment in metastatic breast tumors, while upregulation of hsa-miR-429 is linked to chemosensitivity [67]. In combination with hsa-mir-182 and hsa-mir-96, hsa-mir-183 also serves as a potential prognostic factor for progression-free survival (PFS) and OS [58]. Furthermore, hsa-miR-429 is overexpressed in HER2-positive breast tumors and promotes the proliferation and migration of cancer cells [68]. A recent study performed by Li T et al. (2021), focusing on exosomal hsa-miR-429 in ovarian cancer, showed that this specific miRNA molecule confers resistance to cisplatin via CASR/STAT3 pathway regulation [69].

Upregulation of hsa-mir-190b and hsa-mir-429 have been described to inhibit apoptosis, promoting proliferation and migration in breast cancer cell lines [70]. Previous studies reported that hsa-mir-190b is linked to estrogen-positive breast cancer, correlating with resistance against endocrine therapy [71,72]. hsa-miR-190 was found to target SMAD2 (Mothers against decapentaplegic homolog 2) and suppress metastasis in breast cancer via regulation of TGF-β induced EMT [73]. Yu Y et al. (2019) showed that hsa-miR-190 inhibits the Wnt/β-catenin signaling pathway by targeting the SOX9 transcription factor. This inhibition enhances the sensitivity of endocrine therapy in vivo and in vitro. On the other hand, ZEB1 transcriptionally regulates hsa-miR-190 through promoter binding and competes with ERa signaling, resulting in endocrine therapy resistance [74]. Finally, elevated expression of hsa-mir-3610 has been linked to poor survival in triple-negative breast carcinomas [75]. Collectively, these reports align well with our functional analysis, underlying the importance of our up-regulated signature for breast carcinogenesis.

Focusing on the down-regulated signature, hsa-miR-99a acts as a tumor suppressor, the low expression levels of which are linked to poor outcomes in BRCAs [76]. Elevated levels of hsa-miR-99a reduce the proliferation, migration, and invasion of breast cancer cells. It also targets the *FGFR3* gene, which is involved in mitogenesis and differentiation processes. Downregulation of hsa-miR-99a, as shown in our study, leads to abnormal expression of the *FGFR3* gene, resulting in the activation of PI3K-AKT and RAS/RAF/MEK/MAPK signaling pathways involved in tumor progression [77,78,79]. hsa-miR-337 acts as a tumor suppressor gene in various cancer types. Previous studies have shown that it inhibits proliferation and invasion in cervical cancer by targeting specificity protein 1 (Sp1) and also inhibits colorectal cancer production by interacting with the KRAS/AKT/ERK pathway [80]. hsa-miR-337-3p is a functional product of hsa-miR-337. It was found to target the *ESRP1* gene in breast cancer cells, leading to suppression of migration and invasion. *ESRP1* gene is implicated in cell proliferation and metastasis; thus, down-regulated levels of hsa-miR-337-3p are linked to breast tumor progression [81]. According to a recent study, hsa-miR-1262 acts as an antitumor miRNA that can modulate the expression levels of the low-density lipoprotein receptor (LDLR)-related protein 8 (LRP8), affecting cellular processes such as proliferation, invasion, and migration of breast cancer cells [82]. The aforementioned cellular processes are also modulated in colon cancer cells via NF-κB/miR-1262/FGFR1 interactions [83]. Of note, independent studies reported that hsa-miR-5683 suppresses glycolysis and proliferation in gastric carcinomas [84]. It was also reported that in colon adenocarcinoma patients, the same transcript in combination with five other miRNA molecules could predict overall survival [85].

Last but not least, hsa-miR-10b was shown to be involved in EMT induction. It has been proposed that hsa-miR-10b confers drug resistance to tamoxifen in ER-driven breast tumors by inducing the EMT process and the growth and proliferation of cancer stem cells (CSCs) in vitro and in vivo [86]. Raval et al. (2022) proposed that hsa-miR-10b can predict tumor aggressiveness as it does not correlate to ER/PR status but shows a strong correlation to HER2 status [87]. A research study focusing on miRNAs as potential biomarkers in Lebanese breast cancer patients showed that hsa-miR-10b is downregulated in ER/PR—tumors compared to ER/PR positive tumors [88]. Additionally, hsa-miR-10b is considered a metastamiR and has been demonstrated to be linked to the metastasis process [87]. Taken together, the above reports support our GO results regarding the functions of our selected differentially expressed miRNAs.

## 5. Conclusions and Future Perspectives

In our study, we provide compelling evidence for two miRNA signatures consisting of four up- and five down-regulated miRNAs with strong diagnostic and prognostic efficacy. Moreover, we complement their elevated performance in breast tumors with a multi-cancer analysis across nineteen cancer forms, revealing that both up and down-regulated signatures had a strong preference for breast and other tumors of the female reproductive system.

One limitation of our study refers to the absence of an in vitro or in vivo validation scheme. Another important limitation is the lack of evidence regarding miRNA performance in circulating patient fluids. We are currently working on both research directions, and we plan to independently address both limitations in the near future. Despite these limitations, we believe that our integrated miRNA signatures significantly expand the molecular toolbox of breast cancer detection, representing a well-rounded strategy for assessing miRNAs as diagnostic and prognostic biomarkers. We therefore propose that upon proper clinical validation, both miRNA panels can serve as a functionally validated platform for early detection of breast cancer in a self-confirming, binary fashion (analysis of both signatures in the same sample), with potential extension beyond breast cancer based on the results of our multi-cancer panel analysis.

## Data Availability

Data are contained within the article and Supplementary Materials.

## Supplementary Materials

The following supporting information can be downloaded at: https://www.mdpi.com/article/10.3390/biom14111352/s1, Table S1: Composition of the TCGA BRCA patient cohort; Table S2: Differential expression and statistical analysis of miRNAs in the TCGA BRCA dataset; Table S3: Statistical analysis of selected miRNAs expression in TCGA BRCA stages; Table S4: ROC and cut-off statistical analysis of the up-regulated miRNA signature in TCGA BRCA patients; Table S5: ROC and cut-off statistical analysis of the control miRNA signature in TCGA BRCA patients; Table S6: Statistical analysis of miRNA expression in TCGA BRCA clinical cohorts, Table S7: ROC and cut-off statistical analysis of the down-regulated miRNA signature in TCGA BRCA patients, Table S8: Statistical analysis of miRNA expression in TCGA multi-cancer cohorts organized in organ primaries; Table S9: Gene Ontology analysis of the up-regulated miRNA target genes; Table S10: Gene Ontology analysis of the down-regulated miRNA target genes.
